# Acupuncture decreases amygdala functional connectivity in subjective tinnitus

**DOI:** 10.3389/fneur.2022.986805

**Published:** 2022-12-01

**Authors:** Yating Zhang, Bixiang Zha, Haiping Shi, Ling Cheng, Yinqiu Fan, Wanlin Zhang, Zhihao Rong, Zhaoxing Jin, Nan Gao, Jun Yang, Qingping Zhang

**Affiliations:** ^1^The First School of Clinical Medicine, Anhui University of Chinese Medicine, Hefei, China; ^2^First Affiliated Hospital of Anhui University of Chinese Medicine, Hefei, China; ^3^School of Humanity and International Education and Exchange, Anhui University of Chinese Medicine, Hefei, China; ^4^West China Hospital, Sichuan University, Chengdu, China; ^5^Center for Biomedical Engineering, University of Science and Technology of China, Hefei, China; ^6^School of Acupuncture and Massage, Anhui University of Chinese Medicine, Hefei, China

**Keywords:** acupuncture, subjective tinnitus, functional magnetic resonance imaging, functional connectivity, amygdala

## Abstract

**Introduction:**

Subjective tinnitus is a common and intractable ear disease. The effectiveness of acupuncture in the treatment of subjective tinnitus has been confirmed, but its mechanism of action is not clear. The structures of the amygdala (AMYG) are mainly closely related to emotion in the human brain. This study aimed to investigate the changes in functional connectivity (FC) of AMYG in subjective tinnitus to elucidate the neural mechanism of acupuncture.

**Methods:**

Correlation scale scores of 26 patients with subjective tinnitus were collected, including Tinnitus Evaluation Questionnaire (TEQ), Tinnitus Handicap Inventory (THI) and Visual Analog Scale (VAS). Meanwhile, rs-fMRI data were collected before and after acupuncture treatment in the patients, and in healthy controls (HC) matching the patient's gender and age. Then, AMYG was selected as region of interest to perform FC analysis. Finally, FC patterns of AMYG were first compared between patients with subjective tinnitus and HC, and then within subjects pre-acupuncture and post-acupuncture. Simple linear regression models between correlation scale scores and FC-values were established as well.

**Results:**

Acupuncture treatment relieved the severity of tinnitus. With the acupuncture treatment, the total THI score, TEQ score, and VSA score of patients were significantly lower than before (*p* < 0.05). Compared with HC, FC of tinnitus patients between AMYG and right inferior temporal gyrus and right precuneus significantly decreased before acupuncture (voxel *p* < 0.001, cluster *p* < 0.05, corrected with GRF), while FC of tinnitus patients between AMYG and left superior frontal gyrus and right superior temporal gyrus significantly decreased after acupuncture treatment (voxel *p* < 0.001, cluster *p* < 0.05, corrected with GRF). FC of tinnitus patients between the AMYG and right superior frontal gyrus and left paracingulate gyrus showed significant decrease after acupuncture treatment (voxel *p* < 0.001, cluster *p* < 0.05, corrected with GRF). Besides, the linear regression models of the effect of THI on FC and VAS on FC performed were statistically significant (*p* < 0.05).

**Discussion:**

The findings demonstrate that acupuncture can decrease FC of AMYG, which could be positively correlated with the relief of tinnitus symptoms. This result suggests that acupuncture stimulation can effectively relieve the severity of tinnitus by decreasing FC of AMYG in subjective tinnitus patients.

## Introduction

Subjective tinnitus refers to a type of auditory disease with abnormal sounds in the ear as clinical manifestations only and without any damage to the auditory structure and nervous system of the ear (1). The incidence of tinnitus in the population is relatively high. According to the European Multidisciplinary Tinnitus Guidelines, the prevalence of tinnitus in adults is 10–19%, and about 1/3 of senior citizens have long-term perception of tinnitus (2). The continuous annoying dull chirp will make patients easier angry, damage attention, anxiety and other negative effects. Tinnitus tends to cause emotional disorders, and mental factors affect tinnitus in turn, and the two enter a vicious circle (3). The psychological pressure generated by this vicious circle does serious harm to the physical and mental health of patients (4). Because the symptoms that patients complain about tinnitus are often subjective, and there are few methods for objective assessment, subjective tinnitus has become one of the difficult ontological diseases that need to be solved urgently in clinical practice.

With the development of functional magnetic resonance imaging (fMRI) technology, it is found that the abnormal activity of the auditory system may be caused by changes in functional connectivity (FC) between the auditory network and the non-auditory system (5). Some studies suggest that the central mechanism of subjective tinnitus involves auditory system and non-auditory system (6), tinnitus perception may be the result of auditory-limbic interaction, and structural abnormalities such as amygdala (AMYG) are related to the severity of tinnitus perception (7, 8). AMYG is a part of the limbic system (9), which is involved in a variety of emotional processes (10–12). Chen et al. found that the FC of AMYG to cortical regions was reduced in patients with tinnitus and mood disorders (13). Another study found that the volume of AMYG in tinnitus patients may be related to the distress of tinnitus patients to reduce the related emotions caused by tinnitus (14). On the basis of the above research, we concluded that the amygdala is closely related to tinnitus and speculate whether acupuncture affects AMYG to relieve tinnitus.

Acupuncture is considered to be an effective method for tinnitus and psychiatric diseases (15). At present, acupuncture research based on fMRI mainly includes the relevant research on acupoint specificity (16), acupuncture effect mechanism (17, 18), needling sensation of *deqi* (19, 20) and acupuncture analgesia (21, 22). These studies indicate that the mechanism of acupuncture and the central nervous system may be closely related (23). However, there are few studies on the neural mechanism of acupuncture for subjective tinnitus using fMRI technology. Therefore, this study aims to analyze the effects of acupuncture on AMYG of patients with subjective tinnitus based on fMRI technology, and to further explore the central mechanism of acupuncture in the treatment of subjective tinnitus, whether acupuncture changes the FC of AMYG in patients with subjective tinnitus and whether it is related to clinical symptoms.

## Materials and methods

### Participants

A sample size of twenty-six was decided according to the effective rate of our team treated subjective tinnitus with acupuncture, drop-out rate and other related factors. Twenty-six patients (fourteen females; mean age: 45.2 ± 11.45) with subjective tinnitus and twenty-six age- and gender-matched healthy controls (HC) (fourteen females; age 46.3 ± 12.82) were recruited through the Acupuncture and Rehabilitation Department and the Otolaryngology Department of the First Affiliated Hospital of Anhui University of Chinese Medicine. Exclusion criteria for all participants: (1) history of craniocerebral trauma; (2) those unsuitable for MRI (such as internal metal implants); (3) intracranial lesions such as tumors, hemorrhage and infarction detected after MRI.

This study is a case-control study and approved by the Ethics Committee of the First Affiliated Hospital of Anhui University of Chinese Medicine (ethics number: 2021AH-29). Each subject signed a written informed consent form before participating in the study.

### Evaluation of clinical symptoms

The scales used to assess the severity of tinnitus include Tinnitus Evaluation Questionnaire (TEQ) (24, 25), Tinnitus Handicap Inventory (THI) (26), Visual Analog Scale (VAS) (27). This study used the TEQ independently developed by the Chinese team to score. The TEQ divides the severity of tinnitus into five grades according to the total score of each index with grade one the least severe to grade five the most. The related scales (THI, TEQ, VAS) of subjective tinnitus patients were collected before and after the acupuncture course, respectively.

### Acupuncture treatment

**Figure 1:**
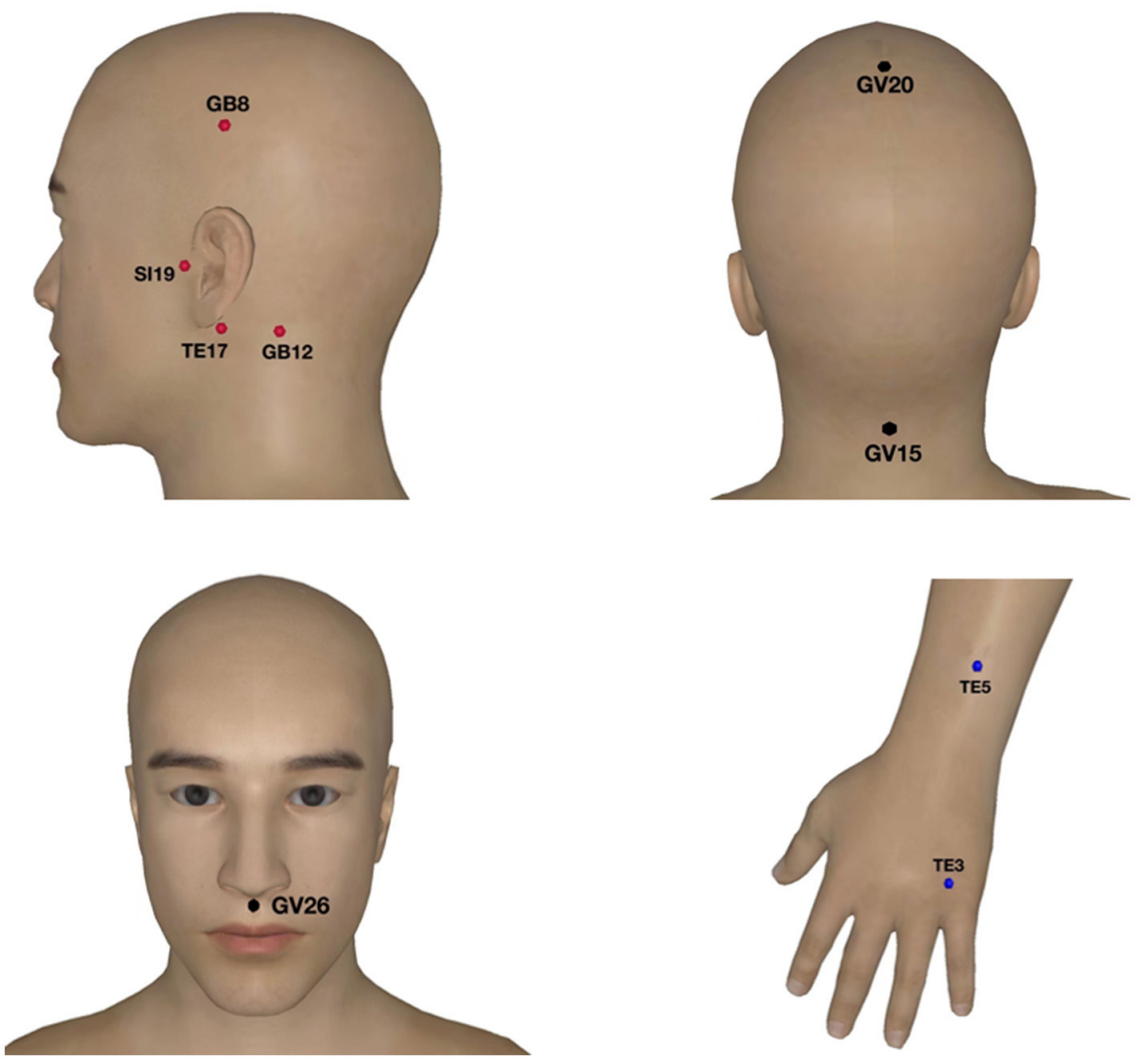
Acupoints selected for acupuncture treatment of subjective tinnitus. The red points are the ones on the affected side of tinnitus, the blue are the distal bilateral acupoints and the black are the Governor Vessel acupoints.

Acupuncture treatment was performed by Professor Yang Jun, the National Famous Doctor of Traditional Chinese Medicine, an honorary title for TCM expert in China. The acupoints on the affected side of tinnitus (SI19, TE17, GB12, GB8), distal bilateral acupoints (TE3, TE5) and the Governor Vessel acupoints (GV20, GV26, GV15) were selected (Figure 1). The acupuncturist inserted sterile and disposable needles (length 40 mm, width 0.35 mm) at the above acupoints. After disinfecting the skin with 75% alcohol, acupuncture a certain depth (1.5–3 cm), the acupuncturist manipulated the needle for obtaining the *deqi* sensation (acid, numbness, swelling and pain). All acupoints were routinely given acupuncture treatment, and SI19 was needle-inserted into with the mouth open. The needles were retained on the acupoints for 30 min, and the patients received 10 times acupuncture treatments (once every other day).

### Data acquirement

The resting-state fMRI (rs-fMRI) scans of all subjects were performed on the same 3.0 T MRI scanner (Discovery MR750, GE, United States) in the Digital Imaging Technology Laboratory of the First Affiliated Hospital of Anhui University of Chinese Medicine, using an 8-channel high-resolution radio-frequency head coil. Data of rs-fMRI for all subjective tinnitus patients as well as HC were collected before and after acupuncture. The data of rs-fMRI after acupuncture was collected 2 days after 10 times of acupuncture treatment. Before scanning, the subjects were instructed to remove metal and magnetic objects from their bodies, and then entered the scanning room after the whole body was relaxed. Earplugs and foam pads were used to reduce noise, and foam pads were added to reduce head movement. During the scanning process, the subjects were instructed to stay awake, close their eyes, lie down quietly, and not to think about anything special.

Structure images were acquired with 3D T1 BRAVO sequence with the following settings: repetition time (TR) = 8.2 ms, echo time (TE) = 3.2 ms, inversion time = 450 ms, flip angle = 12°, field of view = 256 × 256 mm^2^, matrix size = 256 × 256, slice thickness = 1 mm, voxel size =1 × 1 × 1 mm^3^, slice number = 188. Resting-state fMRI data were acquired using a gradient-echo single-shot echo planar imaging sequence, which included 217 time points and took 8 min and 40 s. Specific scan parameters are as follows: TR = 2,400 mms, TE = 30 ms, flip angle = 90°, field of view = 256 × 256 mm^2^, matrix size = 64 × 64, slice thickness = 3 mm, slice number = 46.

### Data processing

Rs-fMRI data processing and analysis were performed with DPABI software (DPABI_V6.1_220101) (28) as follows. Firstly, the image collection data of the first 10 time points were removed to exclude the influence of the initial instability of the magnetic field signal, and at the same time to achieve the subject's adaptation to the magnetic field. Secondly, slice-timing and realignment for head motion correction were performed, and the data with head motion over 3 mm or 3° were excluded. Thirdly, data were co-registered with the structural images, spatial normalized to the Montreal Neurological Institute (MNI) template, and voxels were re-sampled to 3 × 3 × 3 mm^3^ resolution. Finally, data were spatially smoothed with a 6-mm full width at half-maximum (FWHM) Gaussian kernel, and detrended and filtered (0.01-0.08 Hz).

Using WFU_PickAtlas software (http://www.ansir.wfubmc.edu), the bilateral AMYG were used as region of interest (ROI). DPABI software was used to calculate FC between ROIs and the whole brain.

### Statistical analysis

Two-sample *t*-tests were conducted at both pre-acupuncture and post-acupuncture to compare the FC changes between subjective tinnitus patients and HC, age and gender included as covariates. Paired Student's *t*-tests were used to compare the FC changes between pre-acupuncture and post-acupuncture. The results of group analysis were corrected using a Gaussian Random Field (GRF) of voxel *p* < 0.001, cluster *p* < 0.05.

The FC of brain regions with significant differences in subjective tinnitus patients was extracted. Then, simple linear regression models were established by using the clinical characteristics of tinnitus (THI, TEQ, VAS scores) as the independent variables and FC as dependent variable. With the data, six separate simple linear regressions were built which showed the effect of THI on FC, TEQ on FC, and VAS on FC before and after acupuncture, respectively. The significance value was set as *p* < 0.05. Residual normality, homoscedasticity and removal of outliers were checked by using visual inspection of their histograms, P-P plots and scatterplots.

## Results

### Clinical symptoms of tinnitus between pre-acupuncture and post-acupuncture

**Figure 2:**
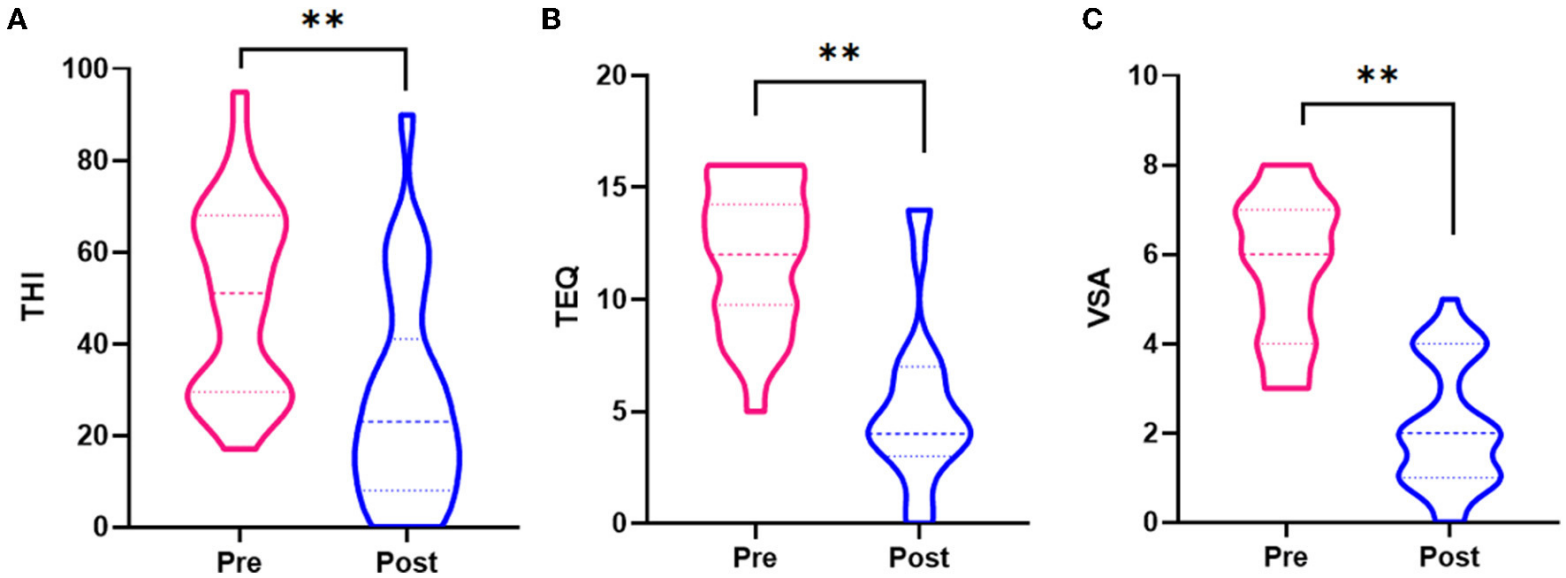
**(A)** Differences of THI scores between pre-acupuncture and post-acupuncture. **(B)** Differences of TEQ scores between pre-acupuncture and post-acupuncture. **(C)** Differences of VAS scores between pre-acupuncture and post-acupuncture. **Significant group difference with *p* < 0.05.

Compared with pre-acupuncture, THI, TEQ, and VAS scores were significantly decreased after acupuncture treatment (Figure 2), and the difference was statistically significant (*p* < 0.05).

### Functional connectivity changes

**Figure 3:**
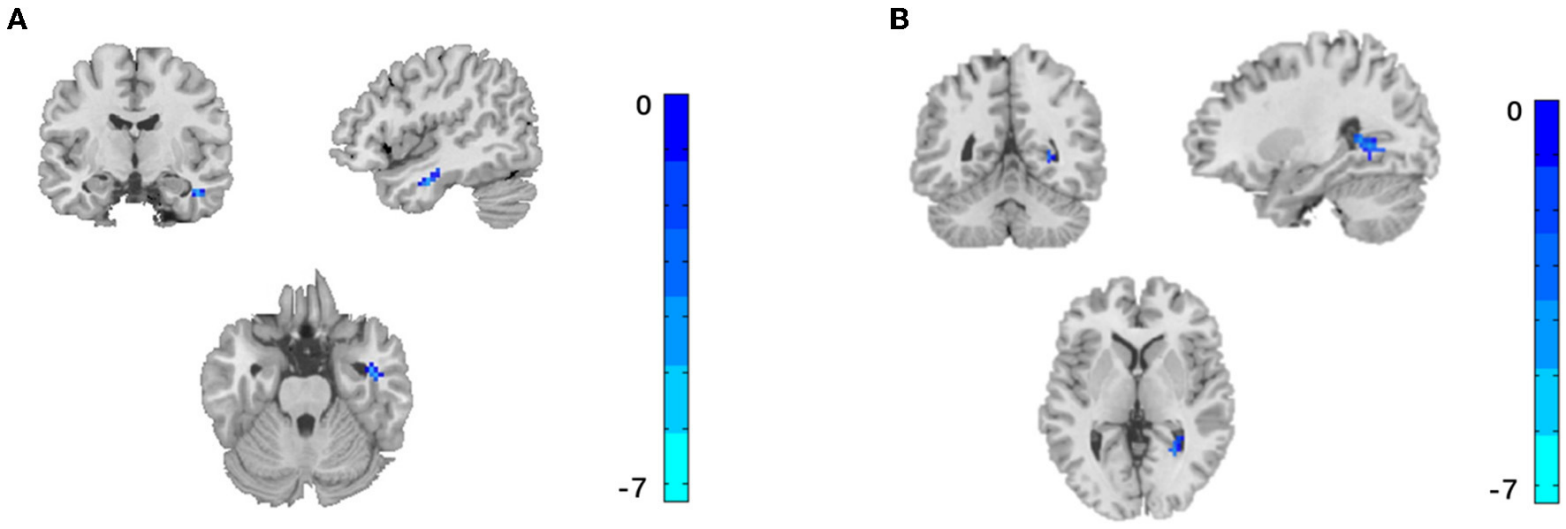
Significant difference for FC between pre-acupuncture in subjective tinnitus and healthy control. The result was corrected for GRF with voxel *p* < 0.001, cluster *p* < 0.05. **(A)** FC between AMYG and right inferior temporal gyrus. **(B)** FC between AMYG and right precuneus.

**Figure 4:**
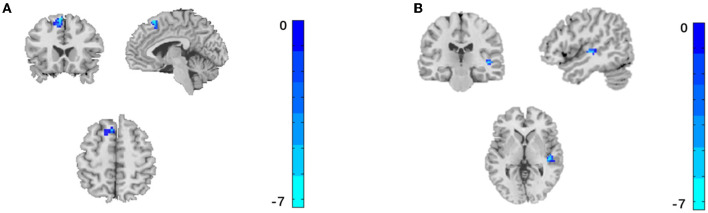
Significant difference for FC between post-acupuncture in subjective tinnitus and healthy control. The result was corrected for GRF with voxel *p* < 0.001, cluster *p* < 0.05. **(A)** FC between AMYG and left superior frontal gyrus. **(B)** FC between AMYG and right superior temporal gyrus.

**Figure 5:**
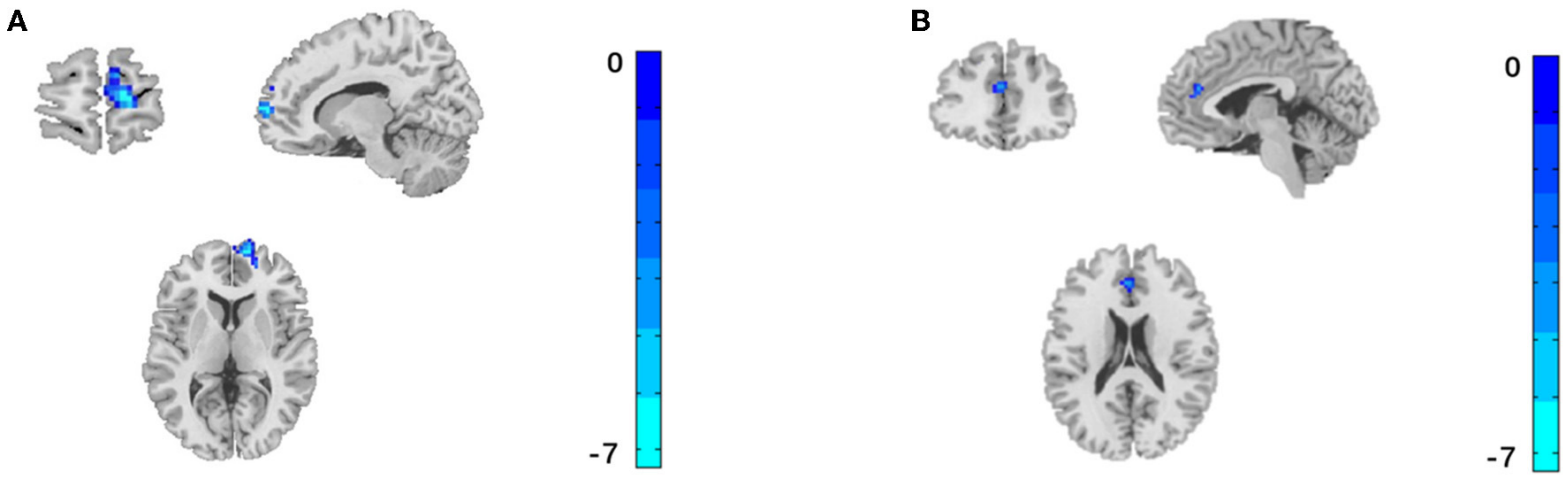
Significant difference for FC between post-acupuncture and pre-acupuncture in subjective tinnitus. The result was corrected for GRF with voxel *p* < 0.001, cluster *p* < 0.05. **(A)** FC between AMYG and right superior frontal gyrus. **(B)** FC between AMYG and left paracingulate gyrus.

Compared with HC, FC between AMYG and right inferior temporal gyrus and right precuneus were significantly decreased at pre-acupuncture (Figure 3 and Table 1, voxel *p* < 0.001, cluster *p* < 0.05, corrected with GRF), and FC between AMYG and left superior frontal gyrus and right superior temporal gyrus were significantly decreased at post-acupuncture (Figure 4 and Table 2, voxel *p* < 0.001, cluster *p* < 0.05, corrected with GRF). Compared with pre-acupuncture, FC between the AMYG and right superior frontal gyrus and left paracingulate gyrus were significantly decreased at post-acupuncture (Figure 5 and Table 3, voxel *p* < 0.001, cluster *p* < 0.05, corrected with GRF).

**Table 1:** Comparison of FC between pre-acupuncture in subjective tinnitus and healthy control.

**Regions**	**Side**	**Voxels**	**MNI**			***T*-value (Peak intensity)**
			**X**	**Y**	**Z**	
**Amygdala**						
Inferior temporal gyrus	R	44	46	−9	−25	−5.599
Precuneus	R	35	27	−50	3	−4.818

The result was corrected for GRF with voxel *p* < 0.001, cluster *p* < 0.05. A positive *T*-value indicates an enhanced FC, and a negative *T*-value indicates a decreased FC.

MNI, Montreal Neurological Institute; Human brain coordinates proposed by the Montreal Neurological Institute.

**Table 2:** Comparison of FC between post-acupuncture in subjective tinnitus and healthy control.

**Regions**	**Side**	**Voxels**	**MNI**			***T*-value (Peak intensity)**
			**X**	**Y**	**Z**	
**Amygdala**						
Superior frontal gyrus	L	47	−7	19	52	−5.255
Superior temporal gyrus	R	42	48	−23	1	−4.995

The result was corrected for GRF with voxel *p* < 0.001, cluster *p* < 0.05. A positive *T*-value indicates an enhanced FC, and a negative *T*-value indicates a decreased FC.

MNI, Montreal Neurological Institute; Human brain coordinates proposed by the Montreal Neurological Institute.

**Table 3:** Comparison of FC between post -acupuncture and pre-acupuncture in subjective tinnitus.

**Regions**	**Side**	**Voxels**	**MNI**			***T*-value (Peak intensity)**
			**X**	**Y**	**Z**	
**Amygdala**						
Superior frontal gyrus	R	99	12	63	12	−6.185
Paracingulate gyrus	L	30	−3	39	21	−4.976

The result was corrected for GRF with voxel *p* < 0.001, cluster *p* < 0.05. A positive *T*-value indicates an enhanced FC, and a negative *T*-value indicates a decreased FC.

MNI, Montreal Neurological Institute; Human brain coordinates proposed by the Montreal Neurological Institute.

### Linear regression between clinical symptoms of tinnitus and FC

**Figure 6:**
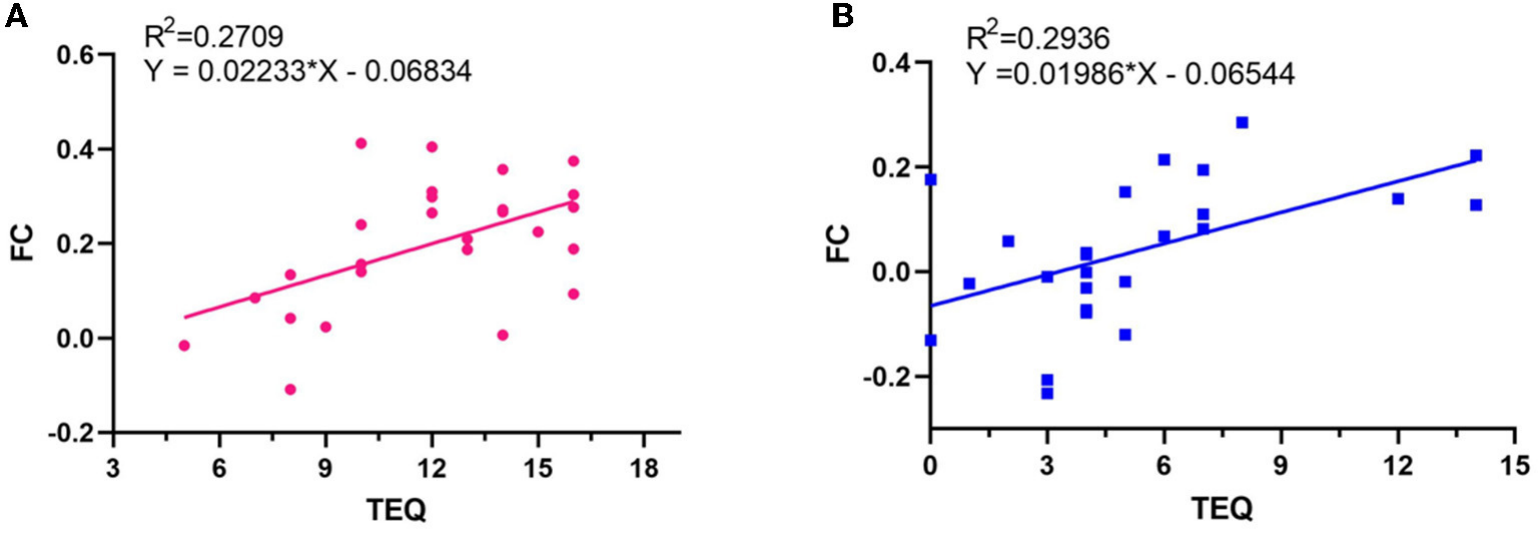
Relationships between FC and TEQ scores. **(A)** At pre-acupuncture. **(B)** At post-acupuncture.

**Figure 7:**
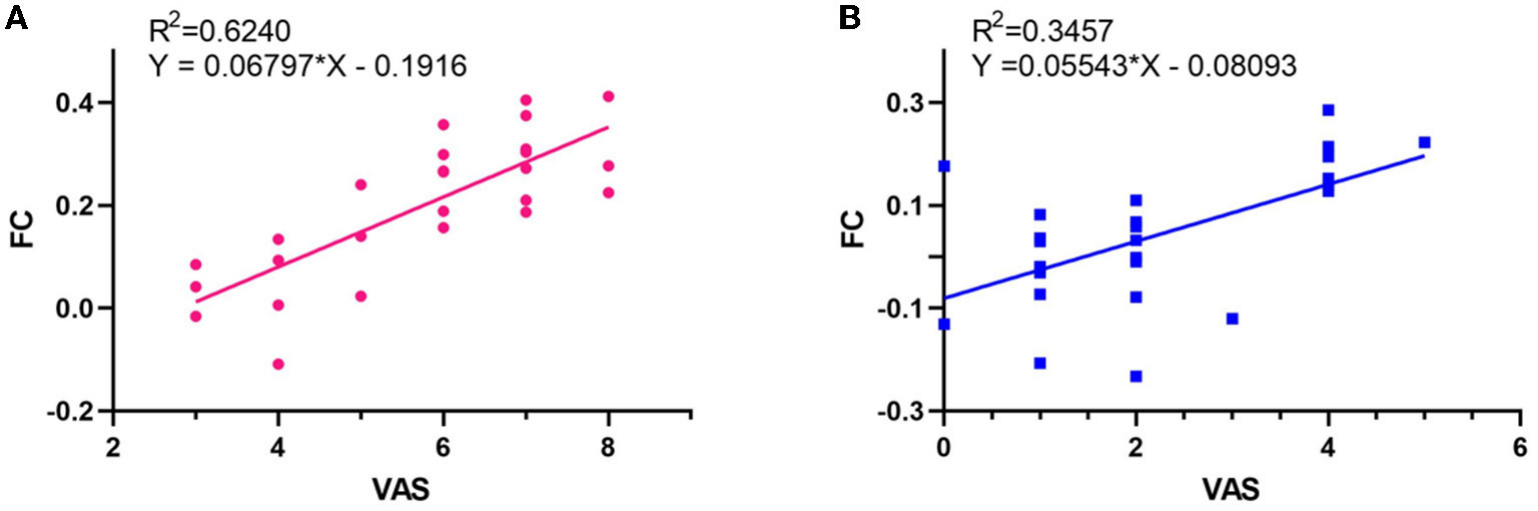
Relationships between FC and VAS scores. **(A)** At pre-acupuncture. **(B)** At post-acupuncture.

All linear regression models performed were statistically significantly (Table 4, *p* < 0.05), except the effect of THI on FC (*p* > 0.05). The linear regression with FC as dependent variable and TEQ as independent variable showed correlation (Figure 6), the determination coefficient (R^2^) of TEQ correlated with the FC was weak and below 50%. The linear regression with FC as dependent variable and VAS as independent outcome showed a strong correlation (Figure 7).

**Table 4:** Linear regressions between clinical symptoms of tinnitus and FC.

	***p*-value**	**b**	**R^2^**	***F*-value**
**At pre-acupuncture**				
TEQ vs. FC	0.006	0.022	0.271	8.917
VAS vs. FC	<0.0001	0.068	0.624	39.830
**At post-acupuncture**				
TEQ vs. FC	0.004	0.020	0.293	9.976
VAS vs. FC	0.001	0.055	0.346	12.680

b, regression coefficient; R^2^, coefficient of determination.

## Discussion

Acupuncture can effectively relieve the severity of tinnitus in patients with subjective tinnitus. This study explored the mechanism of acupuncture in the treatment of subjective tinnitus based on rs-fMRI. The results showed that there are FC of subjective tinnitus patients between AMYG and right inferior temporal gyrus and right precuneus significantly decreased, and acupuncture can decrease FC between AMYG and right superior frontal gyrus and left paracingulate gyrus, which could be positively correlated with the relief of tinnitus symptoms.

This study selected AMYG as ROI and observed the effectiveness of acupuncture on the FC between it and related brain regions, based on the fact that AMYG is one of the most important brain regions involved in emotion recognition and regulation, and it is often studied in relation to emotional disorders (29–31). Our results suggested that compared with HC, FC between AMYG and right inferior temporal gyrus and right precuneus were significantly decreased at pre-acupuncture. It is basically consistent with previous studies that found abnormalities in AMYG (32), inferior temporal gyrus (33) and precuneus (34) in patients with subjective tinnitus. In addition, it is found that the more serious the tinnitus symptoms (TEQ, VAS scores) are, the more FC value of AMYG increases. Based on the above studies, the damaged pattern of AMYG in tinnitus is necessary, and it is speculated that AMYG may participate in the negative emotional regulation related to tinnitus. After acupuncture treatment, FC between the AMYG and right superior frontal gyrus and left paracingulate gyrus were significantly decreased, indicating that acupuncture might be involved in regulating the neuronal activities in the above-mentioned brain regions of tinnitus patients, so as to relieve the perception of tinnitus in patients, but the specific regulation mode is still unclear. However, there are still differences in FC between post-acupuncture and HC, and these are mainly concentrated in the default mode network (precuneus, inferior temporal gyrus, superior temporal gyrus and superior frontal gyrus), which is involved in emotional processing and controls the brain's processing of internal and external environments (35). Therefore, we speculate that the decreased of FC in these brain regions may be the result of self-reactive compensation.

This study reveals part of the central mechanism of acupuncture intervention in tinnitus and provides scientific basis for acupuncture treatment of tinnitus. However, due to the limitation of sample size, it is impossible to grade patients with subjective tinnitus in more details and fully reveal the central mechanism of acupuncture treatment of subjective tinnitus. For further research, the sample size can be increased to score the emotion of patients with subjective tinnitus. The temporal cortex, frontal cortex and cingulate gyrus, which are closely related to subjective tinnitus, could be selected as seed points to analyze and compare the correlation between the spontaneous neural activities in different brain regions and cognition, language and hearing during tinnitus.

## Conclusion

In conclusion, the study has demonstrated there may have decreased FC in the AMYG of patients with subjective tinnitus, and acupuncture may relieve the perception of subjective tinnitus by decreasing the FC of AMYG. Furthermore, this study might provide reference and new idea in clinical practice.

## Data availability statement

The raw data supporting the conclusions of this article will be made available by the authors, without undue reservation.

## Ethics statement

The studies involving human participants were reviewed and approved by the Ethics Committee of the First Affiliated Hospital of Anhui University of Chinese Medicine. The patients/participants provided their written informed consent to participate in this study.

## Author contributions

YZ and BZ wrote the first draft of the article, edited, and revised the article. YZ, NG, and WZ analyzed imaging data. ZR and WZ contributed to data acquisition. YF, ZJ, JY, and QZ helped perform the analysis with constructive discussions. LC revised the language. All authors contributed to and have approved the final version of the article.

## Funding

This work was supported by the National Natural Science Foundation of China (No. 81873370) and Scientific Research Project of Health Commission of Anhui Province in 2021 (AHWJ2021a019).

## Conflict of interest

The authors declare that the research was conducted in the absence of any commercial or financial relationships that could be construed as a potential conflict of interest.

## Publisher's note

All claims expressed in this article are solely those of the authors and do not necessarily represent those of their affiliated organizations, or those of the publisher, the editors and the reviewers. Any product that may be evaluated in this article, or claim that may be made by its manufacturer, is not guaranteed or endorsed by the publisher.
